# The phylogenetic affinities and morphological peculiarities of the bird-like dinosaur *Borogovia gracilicrus* from the Upper Cretaceous of Mongolia

**DOI:** 10.7717/peerj.12640

**Published:** 2021-12-06

**Authors:** Andrea Cau, Daniel Madzia

**Affiliations:** 1 Unaffiliated, Parma, Italy; 2 Department of Evolutionary Paleobiology, Institute of Paleobiology, Polish Academy of Sciences, Warsaw, Poland

**Keywords:** *Borogovia gracilicrus*, Falciphoran condition, Maastrichtian, Mongolia, Paraves, Theropoda, Troodontidae, Upper Cretaceous

## Abstract

*Borogovia gracilicrus* is a small-bodied theropod dinosaur from the Maastrichtian (Upper Cretaceous) Nemegt Formation of southern Mongolia. The taxon is based on a single fragmentary specimen preserving only the distal part of the hindlimbs. The morphology of *Borogovia* shows a peculiar combination of features, some of which are traditionally considered troodontid synapomorphies and others which are unusual for Troodontidae but are shared with other maniraptoran clades. In particular, the second toe of *B. gracilicrus* differs from other troodontids in lacking some of the features which contribute to the specialized ‘sickle-clawed’ second toe, here termed the ‘falciphoran condition’, shared with dromaeosaurids and some other paravians, such as the strongly compressed and falciform ungual. Phylogeny reconstructions intended to explore the affinities of *Borogovia* consistently support its referral within a subclade of troodontids including all Late Cretaceous taxa. The placement of *Borogovia* is not significantly affected by its unusual combinations of hindlimb features or by the homoplasy of the elements forming the falciphoran condition. *Borogovia* is supported as a valid taxon and is distinct from the other Nemegt troodontids, *Tochisaurus* and *Zanabazar*. The lack of a falciform ungual, and the distinctive morphology of the second toe in *B. gracilicrus* are interpreted as a derived specialization among Troodontidae and not as retention of the plesiomorphic condition of non-paravian theropods.

## Introduction

Troodontidae is a species-rich clade of bird-like dinosaurs with unsettled phylogenetic ties and pliable ingroup relationships. Troodontids are traditionally associated with dromaeosaurids within a broader Deinonychosauria (see, *e.g.*, detailed review by Turner, Makovicky & Norell, 2012; Agnolín et al., 2019; Pittman et al., 2020). The name Deinonychosauria, anchored on the dromaeosaurid taxon *Deinonychus*, explicitly refers to the ‘*δɛινóς őνυξ*’ (Ostrom, 1969), the specialized second toe bearing a large, sickle-shaped, trenchant ungual, variably present in dromaeosaurids and troodontids (Turner, Makovicky & Norell, 2012). Paul (1988a) extended the meaning of the morphological term ‘sickle-claw’ using it as a synecdoche for ‘dromaeosaurid’ (Paul, 1988a). The same author then introduced ‘sickle-clawed’ (Paul, 1988b) as an informal term for dromaeosaurids, troodontids and other close relatives of birds, taxa that are now all inferred to be paravians (Sereno, 1997; Sereno, 1998).

Nevertheless, the phylogenetic hypotheses comprising rootward paravian interrelationships remain conflicting and particularly sensitive to taxon and character samplings (see *e.g.*, Turner, Makovicky & Norell, 2012; Agnolín & Novas, 2013; Brusatte et al., 2014; Cau, Brougham & Naish, 2015; Cau et al., 2017; Cau, 2018; Hartman et al., 2019; Pei et al., 2020; Pittman et al., 2020). As such, they represent a subject for further studies exploring, among other things, assessments of character distribution and use of a wider array of analytical methods.

**Figure 1:**
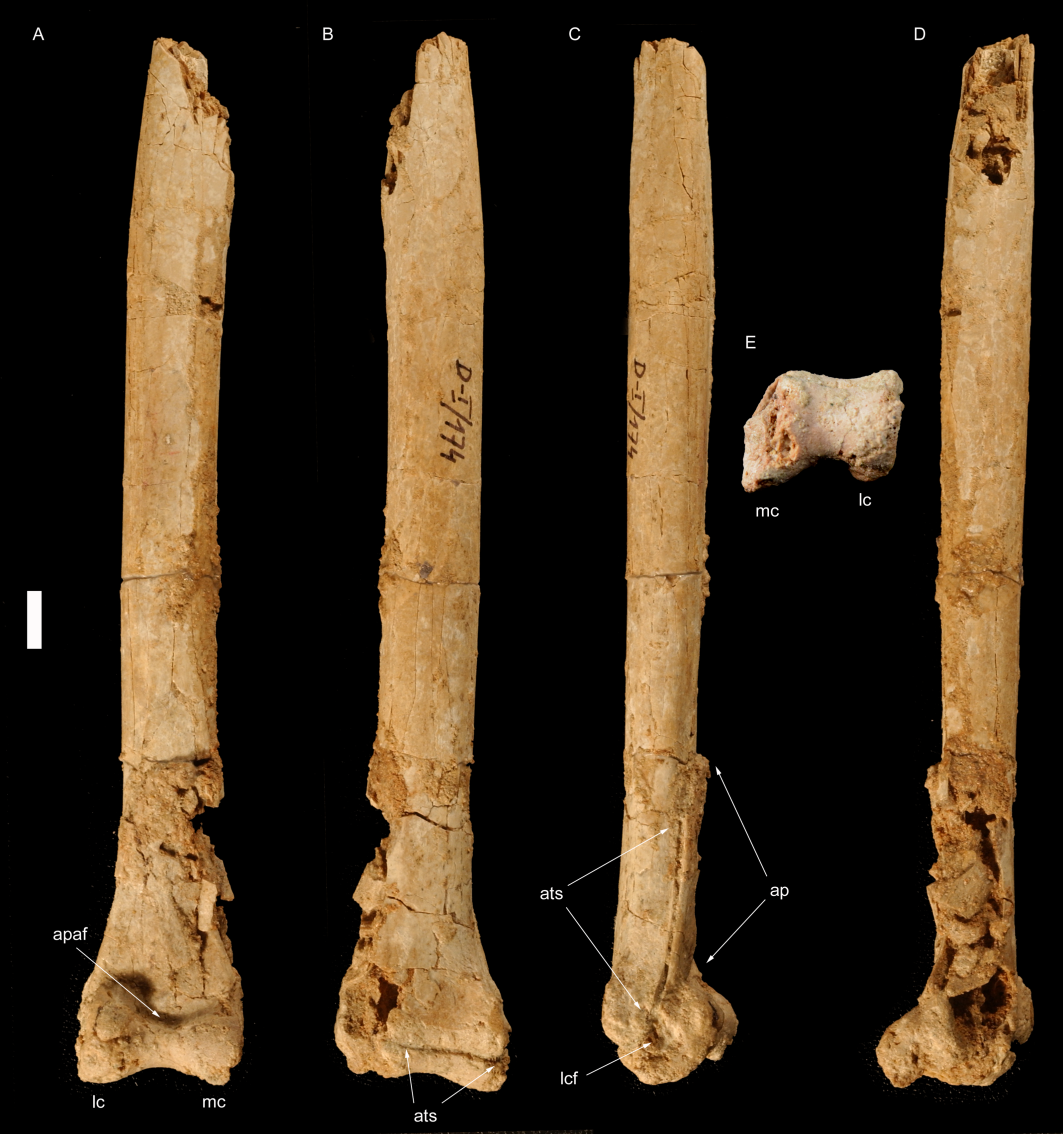
Right tibiotarsus of *Borogovia gracilicrus* ZPAL MgD-I/174. (A) Extensor view; (B) flexor view; (C) lateral view; (D) medial view; (E) distal view. Abbreviations: apaf, fossa on ascending process of astragalus; ats, astragalus-tibia suture; lc, lateral condyle; mc, medial condyle. Scale bar = 1 cm. Photograph credits: Daniel Madzia.

**Figure 2:**
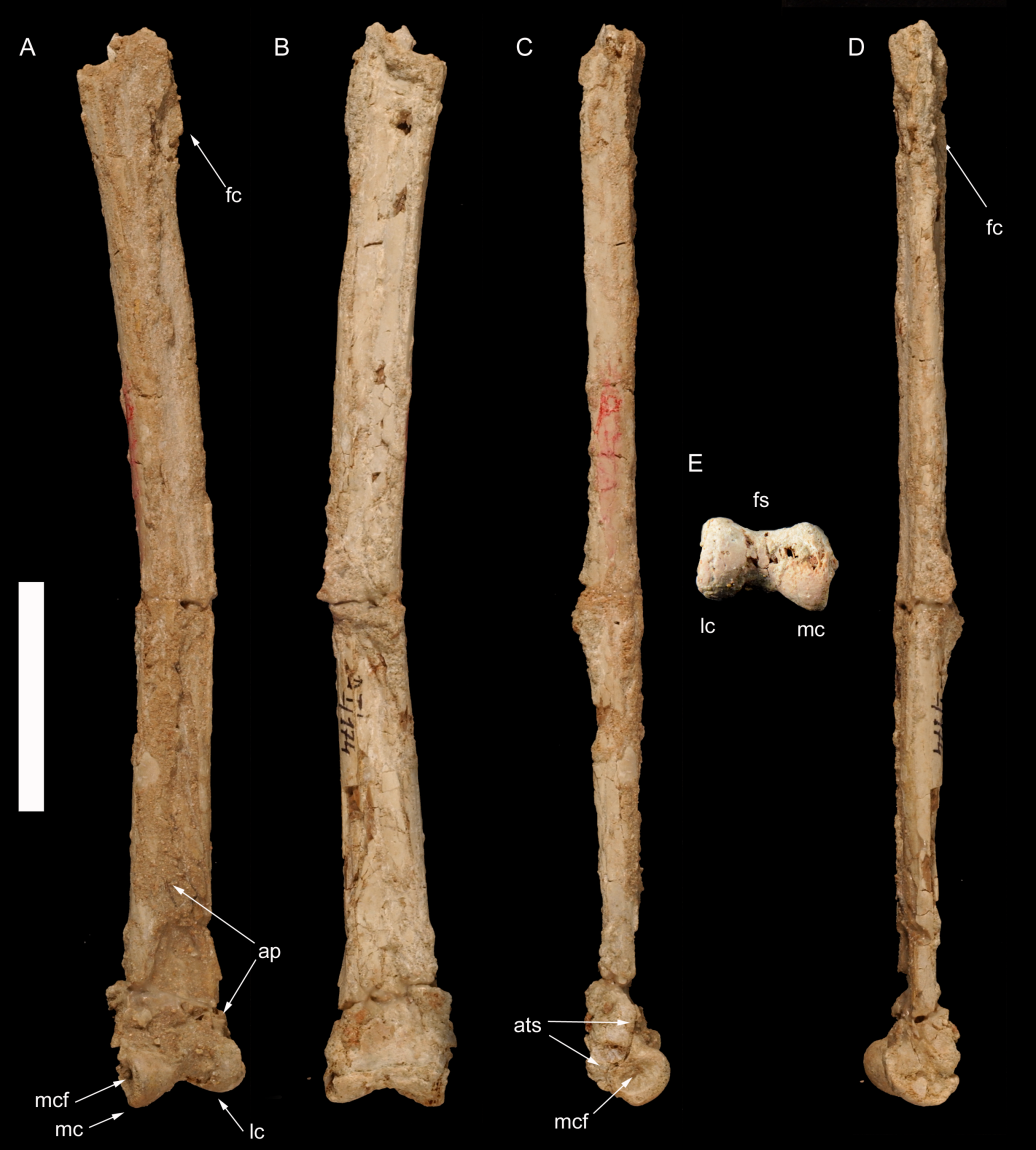
Left tibiotarsus of *Borogovia gracilicrus* ZPAL MgD-I/174. (A) Extensor view; (B) flexor view; (C) medial view; (D) lateral view; (E) distal view. Abbrevations: ap, ascending process; fc, distal end of fibular crest; fs, flexor sulcus; lc, lateral condyle; mc, medial condyle; mcf, fossa on medial side of medial condyle. Scale bar = 4 cm. Photograph credits: Daniel Madzia.

**Figure 3:**
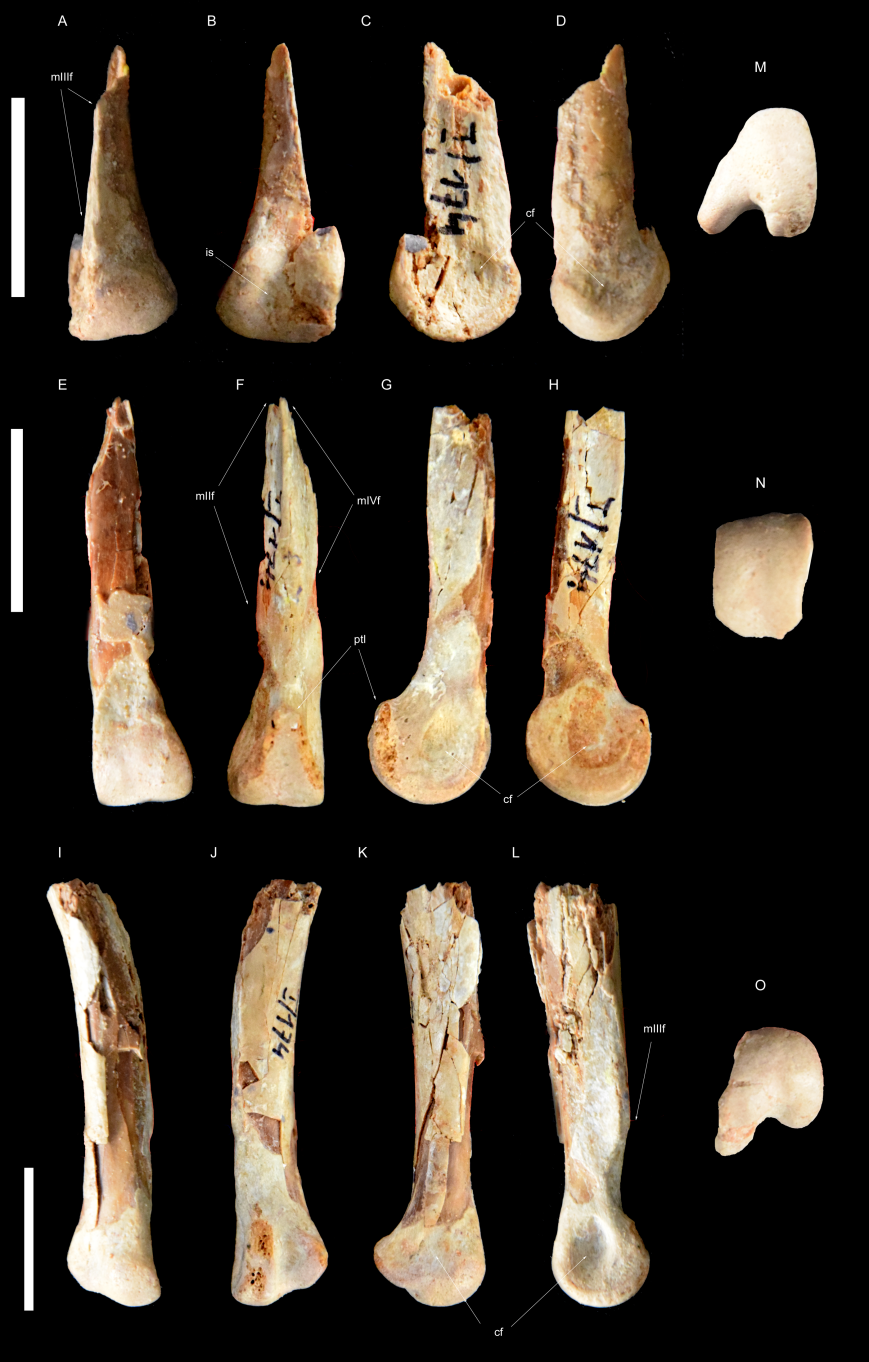
Right metatarsus of *Borogovia gracilicrus* ZPAL MgD-I/174. (A–D, M) Distal end of metatarsal II; (E–H, N) distal end of metatarsal III; (I–L, O) distal end of metatarsal IV. (A, E, I) Extensor view: (B, F, J) flexor view; (C, G, K) lateral view; (D, H, L) medial view; (M, N, O) distal view. Abbreviations: cf, condylar fossa; is, intercondylar sulcus; mtIIf, facet for metatarsal II; mtIIIf, facet for metatarsal III; mtIVf, facet for metatarsal IV; ptl, posterior tongue-like margin. Scale bars = 2 cm. Photograph credits: Daniel Madzia.

**Figure 4:**
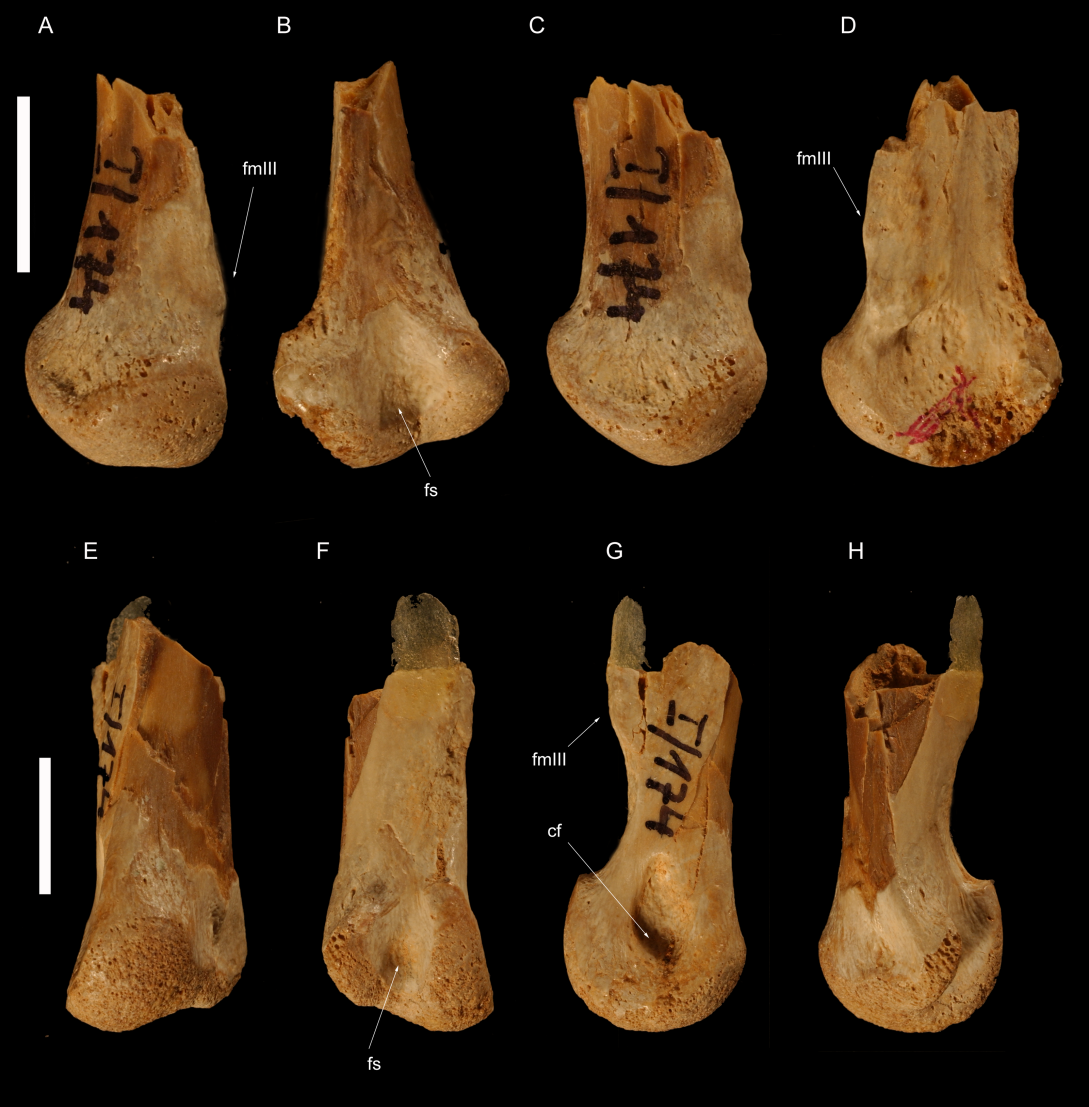
Left metatarsus of *Borogovia gracilicrus* ZPAL MgD-I/174. (A–D) Distal end of metatarsal II; (E–H) distal end of metatarsal IV. (A, E) extensor view; (B, F) flexor view; (C, G) medial view; (D, H) lateral view. Abbreviations: cf, condylar fossa; fmtIII, facet for metatarsal III; fs, flexor sulcus. Scale bars = 1 cm. Photograph credits: Daniel Madzia.

**Figure 5:**
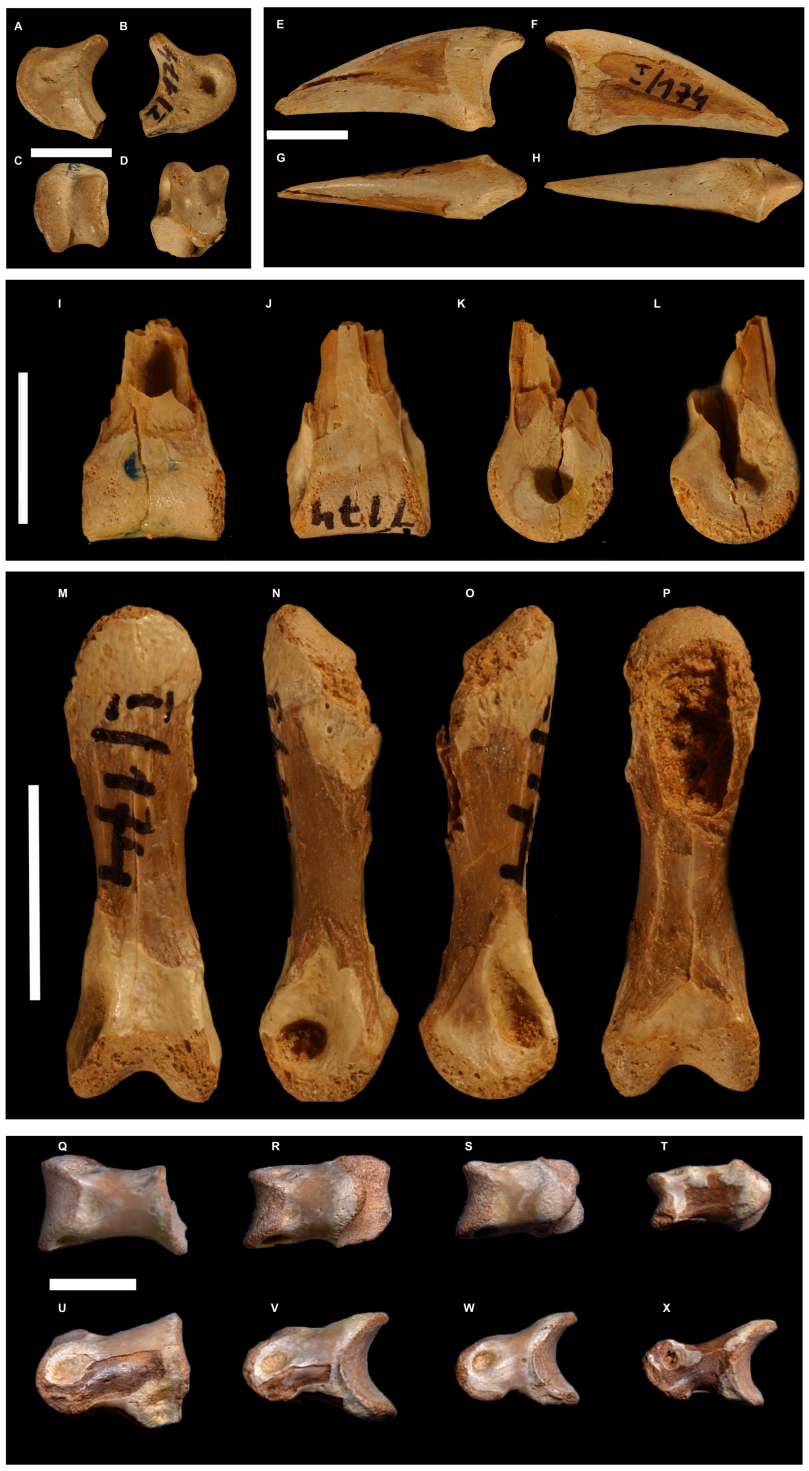
Selected toe phalanges of *Borogovia gracilicrus* ZPAL MgD-I/174. (A–D) Phalanx II-2 of right toe; (E–H) phalanx II-3 of right toe; (I–L) phalanges III-1 of right toe; (M–P) phalanx III-3 of right toe; (Q–X) phalanges IV1-4 of left toe. (B, F, L, O) lateral view; (A, E, L, K, N) medial view; (C) distal view; (I, P) flexor view; (D, G, J, M) extensor view. Scale bars = 1 cm. Photograph credits: Daniel Madzia.

Here we provide new information on the peculiar morphology of *Borogovia gracilicrus* Osmólska, 1987, a troodontid from the Maastrichtian (Upper Cretaceous) Nemegt Formation of southern Mongolia. The only known specimen of *B. gracilicrus* is a partial skeleton, represented exclusively by distal hindlimb elements (Figs. 1–5). The material was obtained during the 1971 Polish-Mongolian paleontological expedition to the Nemegt Basin. It was discovered at the base of the Altan Uul sections, at Altan Uul IV, which exposes the lower Nemegt Formation (Eberth, 2018). Osmólska (1987) referred *Borogovia* to Troodontidae based on the morphology of the metatarsus, which shares with other troodontids, among other features, the combination of metatarsal III being pinched proximally and wedged between the adjacent metatarsals, and a transversely robust metatarsal IV which forms more than half the width of the metatarsus.

The troodontid affinities of *Borogovia* have never been questioned; despite having been tested in only a few numerical analyses. Although these analyses supported the original taxonomic attribution of *Borogovia*, they did not resolve the precise placement of the taxon among troodontids (*e.g.*, extended data figure 9 in Cau et al., 2017; supplementary figure 1 in Hartman et al., 2019). Indeed, even though the combination of apomorphies in the metatarsus of *Borogovia* strongly supports referral to Troodontidae, this taxon differs from all other troodontids and paravians in having an unusual set of features present in the toes (Osmólska, 1987), including the non-falciform shape of the second ungual.

The specialized second toe, bearing a sickle-like ungual, which is absent in *Borogovia*, has frequently been considered a key synapomorphy of a clade including troodontids and dromaeosaurids and usually referred to as Deinonychosauria (Colbert & Russell, 1969; Russell, 1969; Sereno, 1998; Turner, Makovicky & Norell, 2012). Osmólska (1987) compared the feet of dromaeosaurids and troodontids, concluded that the specialized second toe might have developed convergently in the two groups, and dismissed the possibility of a monophyletic Deinonychosauria. The hypothesis that the specialized second toe in troodontids evolved independently from the condition in dromaeosaurids was further discussed by Currie & Peng (1993). Yet, after the introduction of phylogenetic methods in theropod systematics, Deinonychosauria has been frequently reconstructed as a clade (see review in Turner, Makovicky & Norell, 2012), with its monophyly supported by synapomorphies pertaining to the skull and axial skeleton, rather than only by the shared presence of the ‘sickle-clawed’ second toe (Turner, Makovicky & Norell, 2012; Brusatte et al., 2014; Hartman et al., 2019; Pei et al., 2020; Pittman et al., 2020). Alternatively, this grouping has been considered as a paraphyletic series of successive sister taxa to avialan (bird-line) paravians, and the evolution (and secondary loss) of the specialized second toe has been interpreted to be homoplastic within Paraves (*e.g.*, Agnolín & Novas, 2013; Cau, Brougham & Naish, 2015; Cau et al., 2017). These alternative results suggest that a specialized second toe bearing a falciform ungual (Ostrom, 1969), here termed the ‘falciphoran condition’ (from Latin ‘*falcis*’, sickle, and Greek ‘*φoρɛας*’, bearer), did not necessarily originate only once and was not necessarily associated exclusively with Deinonychosauria (here meant as the troodontid-dromaeosaurid grouping, and excluding avialans and other theropods; following Sereno, 1998; Turner, Makovicky & Norell, 2012).

Owing to the incomplete knowledge of the morphological features present in *Borogovia gracilicrus*, the primary aims of this study are (1) to supplement the original description of Osmólska (1987), in part by providing updated discussion of some morphological peculiarities observable in *B. gracilicrus* and comparisons with the numerous paravian taxa erected over the last three decades, and (2) to test and discuss the phylogenetic affinities of this unusual taxon.

## Material and Methods

### Material

The study is based on personal examination of the type specimen of the paravian theropod *Borogovia gracilicrus* consisting of an incomplete pair of hindlimbs. The material is housed at the Institute of Paleobiology of the Polish Academy of Sciences under the catalog number ZPAL MgD-I/174.

### Phylogenetic analysis

The phylogenetic affinities of *Borogovia* were investigated using a data matrix, modified from Cau et al. (2017) and Cau (2020), and focused on pennaraptoran coelurosaurs. Several recently-described troodontid taxa, absent in previous iterations of this data set (*e.g.*, *Almas*, *Daliansaurus*, *Hesperornithoides*, *Jianianhualong*, and *Liaoningvenator*; Pei et al., 2017; Shen et al., 2017a; Shen et al., 2017b; Xu et al., 2017; Hartman et al., 2019) were included. The content and inclusiveness of the Operational Taxonomic Unit (OTU) ‘*Troodon*’ is debated (*e.g.*, Van der Reest & Currie, 2017; Cullen et al., 2021). Here, we follow Cullen et al. (2021) in treating *Latenivenatrix* as a junior synonym of *Stenonychosaurus*. The names ‘*Troodon*’ and ‘*Troodon formosus*’ are provisionally restricted to the type material only (an isolated tooth crown, Leidy, 1856; Currie, 1987; Van der Reest & Currie, 2017), pending an explicit assessment of its synonymy with other named North American troodontids. Although the *Troodon* OTU comprises a largely uninformative string of codings, which is redundant with subsets of scores from other included OTUs (*i.e., Saurornithoides*, *Zanabazar*), the taxon name *Troodon* is eponymous for the clade names Troodontidae and Troodontinae, and acts as an ‘anchor’ for the phylogenetic definitions of the two clade names. Therefore, the *Troodon* OTU has been included in a preliminary analysis to test (1) its placement relative to other maniraptorans and (2) the monophyly and inclusiveness of the taxonomic groups anchored on *Troodon*.

Two isolated teeth, respectively from the Upper Jurassic Morrison Formation of the USA (the holotype of *Koparion douglassi*, Chure, 1994) and the Upper Cretaceous Kallamedu Formation of India (Goswami et al., 2013), have been referred to Troodontidae. Although these two specimens might significantly expand the stratigraphic and geographic distribution of Troodontidae, we note that the features supporting these referrals are widespread among archosauriform teeth (*e.g.*, Prasad & De Lapparent de Broin, 2002) and are not exclusive to troodontids (Agnolín et al., 2019). In particular, based on overall similarity with teeth of notosuchian and atoposaurid crocodylomorphs, taxa which are known from the two aforementioned formations (*e.g.*, Prasad & Lapparent de Broin, 2002; Lauprasert et al., 2011; Puértolas-Pascual, Rabal-Garcés & Canudo, 2015), we cannot dismiss the hypothesis that these specimens are not of theropod origin. Therefore, they are provisionally excluded from our analyses.

The data matrix (170 OTUs *vs* 1830 character statements) was analysed following the protocol described by Madzia & Cau (2017), using parsimony as the search criterion. The inferred topologies resulting from these analyses were compared to visualize the effects of the use of different weighting settings on paravian basal topology (*e.g.*, deinonychosaur monophyly), troodontid inclusiveness and troodontid ingroup relationships. Parsimony analyses were performed in TNT 1.5 (Goloboff, Farris & Nixon, 2008): we performed 100 ‘New Technology’ heuristic search analyses using default settings, followed by exploration of the tree islands found, storing 50,000 trees per replication. We performed two types of analyses: (1) with all characters having equal weight, and using the ‘Quick pruning heuristic’ function in TNT 1.5 to identify the ‘wildcard’ OTUs (the latter were removed to improve the resolution of the strict consensus of the shortest topologies reconstructed); (2) using the ‘Extended Implied Weighting’ option of TNT 1.5 (Goloboff, 1993; Goloboff, 1995; Goloboff, Farris & Nixon, 2008) with four runs performed (setting the concavity parameter *K* as alternatively = 3, 9, 15, and 27) and the ‘Extended Weighting’ function using default settings.

## Systematic Paleontology

**Table:** 

Theropoda Marsh, 1881 [Naish et al., 2020]
Paraves Sereno, 1997
Troodontidae Gilmore, 1924
*Borogovia gracilicrus* Osmólska, 1987

**Type locality.** Altan Uul IV, lower Nemegt Formation; Maastrichtian, Upper Cretaceous; Mongolia.

**Holotype.** ZPAL MgD-I/174, both tibiae missing most of their proximal ends (Figs. 1 and 2), fragment of proximal fibula, almost complete astragalocalcanea fused to the tibiae (Figs. 1 and 2), both feet incompletely preserved (of the left pes: distal parts of metatarsals II and IV, second toe with phalanx II-2 damaged, third toe including only distal end of phalanx III-1 and slightly damaged phalanx III-3, almost complete fourth toe with proximal part of ungual; of the right pes: distal portions of articulated metatarsals II, III and IV, complete second toe, but phalanx II-2 lacking ventrodistal ‘heel’, third toe with preserved distal end of phalanx III-1, phalanx III-2 dorsodistally with some damage and phalanx III-3 ventrodistally with some damage, ungual with damaged dorsal edge, complete fourth toe with ungual somewhat damaged at the dorsal edge; Osmólska, 1987) (Figs. 3–5).

**Diagnosis** (emended from Osmólska, 1987). Troodontid theropod with the following combination of features (autapomorphies marked by *): distinct semilunate fossa at base of ascending process of astragalus; lateral condyle of astragalocalcaneum extends farther proximally across extensor surface of bone than medial condyle; extensor surface of the distal end of the first phalanx of second toe moderately developed, not significantly expanded dorsally (along the flexor-extensor axis) relative to the shaft; second phalanx of second toe extremely abbreviated (length subequal to proximal depth) and lacking a distinct constricted diaphysis between the articular ends; penultimate phalanx of third toe subequal (96%) in length to preceding phalanx; phalanges of fourth toe more robust than those of third toe*; ungual of second toe low, unrecurved and non-falciform*; and ventral margins of unguals II-IV straight in lateral/medial view.

**Differential diagnosis (with respect to other Nemegt Formation troodontids).** *Borogovia gracilicrus* differs from *Tochisaurus nemegtensis* (Kurzanov & Osmólska, 1991) in that the distal end of metatarsal II is subequal in width to the distal ends of metatarsals III and IV (in *Tochisaurus*, the distal end of metatarsal II is 75% as wide as the distal ends of metatarsals III and IV; see table 1 in Kurzanov & Osmólska, 1991). Furthermore, the shallow proximodistal sulcus running along the flexor surface of the distal end of metatarsal III of *Tochisaurus nemegtensis* (Kurzanov & Osmólska, 1991) is absent in *B. gracilicrus*. *Borogovia* differs from *Zanabazar junior* (Norell et al., 2009) in the following combination of features: the ascending process of the astragalus lacks a distinct bump in the middle of the extensor surface (present in *Zanabazar*, Norell et al., 2009); the medial condyle of the astragalus is not buttressed by a swollen area (present in *Zanabazar*, Norell et al., 2009); the long axis of the medial fossa of the medial astragalar condyle forms a wider angle with the proximodistal axis of the tibia (about 60°, compared to an angle of less than 30° in *Zanabazar*, see fig. 33B in Norell et al., 2009); in extensor view, the lateral condyle of the astragalus is more extended proximally than the medial condyle (in *Zanabazar*, the two condyles are comparably extended proximally).

**Remarks.** *Borogovia* shows a unique combination of features which supports the validity of this taxon and distinguishes it from other Cretaceous theropods. Although the majority of these features also supports a paravian and troodontid placement for this taxon, some are unusual for a paravian, and are shared with other maniraptoriform clades.

Osmólska (1987) listed the very slender and long tibiotarsus among the diagnostic features of *Borogovia gracilicrus*. Although we agree that the tibiotarsus of this theropod is noteworthy for its elongation and slenderness, the diagnostic status of this feature is problematic. The tibiotarsi of *B. gracilicrus* are both incomplete, and their actual length could only be inferred to be at least ten times their distal mediolateral width (Figs. 1 and 2). This ratio is expected to be ontogenetically variable and allometrically-controlled, making any explicit definition of that feature that might be used in the species diagnosis questionable without a sufficiently large sample. Furthermore, the lack of complete tibiotarsi in several troodontids, including the other two Nemegt taxa (*e.g.*, Kurzanov & Osmólska, 1991; Norell et al., 2009; Zanno et al., 2011) prevents any unambiguous differentiation between the peculiar elongation of the tibiotarsi in *B. gracilicrus* and the condition seen in its closest relatives*.*

In *Borogovia*, the suture between the astragalus and calcaneum is obliterated, and the two bones are tightly sutured to the tibia (Figs. 1 and 2). Yet, the ascending process of the astragalus can be distinguished from the overlapped tibia along its medial margin, and can be seen to stand out distinctly from the tibial shaft in medial view. This combination of features has been considered a paravian synapomorphy (Turner, Makovicky & Norell, 2012). In the majority of other coelurosaurs, the suture between the astragalus and calcaneum is not obliterated, and the ascending process is not tightly sutured to the tibia (*e.g.*, *Gallimimus*, Osmólska, Roniewicz & Barsbold, 1972; *Tyrannosaurus*, Brochu, 2003; *Khaan*, Balanoff & Norell, 2012). In many avialans, the degree of coossification of the tibiotarsal elements is more extreme than in other paravians, including *Borogovia*, and the ascending process of the astragalus can barely, if at all, be distinguished from the tibia in adult individuals (*e.g.*, *Hollanda*, Bell et al., 2010; *Qiliania*, Ji et al., 2011).

The ascending process of the astragalus of *Borogovia* is expanded proximally, and overlaps the whole extensor surface of the distal end of the tibia (Figs. 1 and 2). An ascending process of the astragalus which is more than twice as long proximodistally than wide at its base, covering the whole distal shaft of the tibia, is a condition widespread among maniraptoriforms (*e.g.*, *Velociraptor*, Norell & Makovicky, 1999). This feature distinguishes *Borogovia* from some parvicursorine alvarezsaurids, in which the astragalus bears a proportionally narrower ascending process of the astragalus, restricted to the medial half of the tibiotarsal complex and having a concave lateral margin (*e.g.*, *Mononykus*, Perle et al., 1994).

The distal end of the tibiotarsus of *Borogovia* bears prominent condyles which describe a broad arch in both the extensor and distal directions. In distal view, the flexor margin of the astragalocalcaneum describes a broad concavity (extension of the intercondylar sulcus onto the flexor surface), bordered by the projections of the condyles (Figs. 1E, 2E). In most theropods, the flexor margin of the distal end of the astragalocalcaneum is more flattened, lacking both a distinct flexor projection of the condyles and an extension of the intercondylar sulcus onto the distal end of the flexor surface (*e.g.*, Osmólska, Roniewicz & Barsbold, 1972; Ostrom, 1969; Brochu, 2003). A condition comparable to *Borogovia* is present in avialans (*e.g.*, Bell et al., 2010).

As preserved, the shafts of both metatarsals II in *Borogovia* are markedly compressed transversely, making them less than half as wide as the distal articular surfaces of the same bones (Figs. 3 and 4). This condition differs from the majority of coelurosaurs, regardless of their body size, which usually have a more proportionally robust diaphysis of metatarsal II (*e.g.*, *Deinonychus*, Ostrom, 1969; *Tyrannosaurus*, Brochu, 2003, *Hulsanpes*, Cau & Madzia, 2018). The diaphysis of metatarsal II is significantly narrower than the distal end of the bone in Late Cretaceous troodontids (*e.g.*, *Gobivenator*, Tsuihiji et al., 2014; *Philovenator*, Currie & Peng, 1993); *Talos*, Zanno et al., 2011).

The distal end of metatarsal II of *Borogovia* is uniformly convex in extensor view, because the flexor sulcus does not extend onto the distal and extensor surfaces of the bone (Figs. 3, 4). This condition is widespread among coelurosaurs (*e.g.*, *Gallimimus*, Osmólska, Roniewicz & Barsbold, 1972; *Talos*, Zanno et al., 2011; *Tyrannosaurus*, Brochu, 2003) and differs from most dromaeosaurids (*e.g.*, *Velociraptor*, Norell & Makovicky, 1999) and some avialans (*e.g.*, *Yungavolucris*, Chiappe, 1993), which show a ginglymous distal end of metatarsal II and a distinct sulcus running along the extensor surface of the distal end of the bone.

When articulated, the distal ends of metatarsals II to IV of *Borogovia* closely fit together along well-defined surfaces. In particular, the distal shaft of metatarsal III is triangular in cross section, being tightly wedged between articular facets on the shafts of the other two metatarsals, with the latter broadly overlapped by the former in extensor view (Holtz, 1994; Snively, Russell & Powell, 2004). This feature is one of the components of the ‘arctometatarsalian condition’ (Holtz, 1994), and is widespread among Late Cretaceous coelurosaurs, including tyrannosaurids (Snively, Russell & Powell, 2004), ornithomimids (Holtz, 1994; Snively, Russell & Powell, 2004), parvicursorine alvarezsaurids (Perle et al., 1994), some oviraptorosaurs (*i.e.,* caenagnathids and avimimids; Snively, Russell & Powell, 2004; Funston et al., 2016), and late-diverging troodontids (Kurzanov & Osmólska, 1991; Snively, Russell & Powell, 2004; Norell et al., 2009; Tsuihiji et al., 2014). Among the gracile-limbed theropods from the Upper Cretaceous of Mongolia, the metatarsal III morphology of *Borogovia* distinguishes this taxon from eudromaeosaurs (Ostrom, 1969; Holtz, 1994) and halszkaraptorines (Cau & Madzia, 2018), in both of which the distal shaft has a quadrangular cross section and is not wedged between the adjacent metatarsals. In those dromaeosaurids, metatarsal III barely, if at all, overlaps the distal shafts of the other metatarsals in extensor view (*e.g.*, Snively, Russell & Powell, 2004; Cau & Madzia, 2018).

Related to the previous feature, although not strictly co-variant with it, is the presence of flanges along the dorsal margins of the distal shaft of metatarsal III which overlap the adjacent metatarsals (Snively, Russell & Powell, 2004). This feature is particularly evident in ornithomimids (Osmólska, Roniewicz & Barsbold, 1972; Holtz, 1994; Snively, Russell & Powell, 2004), avimimids (Funston et al., 2016) and parvicursorines (Perle et al., 1994; Turner, Nesbitt & Norell, 2009), which show a distinct and symmetrical development of these flanges over both metatarsals II and IV. *Borogovia* differs from the aforementioned taxa in that the flanges of metatarsal III are less symmetric and more poorly developed. In particular, the lateral margin of the distal half of metatarsal III of *Borogovia*, adjacent to metatarsal IV, describes a straight line in extensor view, parallel to the proximodistal axis of the bone (Fig. 3E). This feature distinguishes troodontids from other arctometarsalian taxa (*e.g.*, ornithomimids, Snively, Russell & Powell, 2004).

In the majority of theropods, the trochlear distal articular surface of metatarsal III extends onto the flexor surface and bears a longitudinal sulcus (*e.g.*, *Gallimimus*, Osmólska, Roniewicz & Barsbold, 1972; *Mononykus*, Perle et al., 1994). The third metatarsal of *Borogovia* shares a peculiar condition with *Sinornithoides* (Russell & Dong, 1993) and a specimen referred to *Stenonychosaurus* (Russell, 1969), in that the flexor surface of the distal end is subtriangular in flexor view and lacks a distinct longitudinal sulcus (Fig. 3F). This ‘tongue-like’ plantar extension of the articular surface, devoid of a longitudinal sulcus, is only incipiently developed in *Tochisaurus* (Kurzanov & Osmólska, 1991) and absent in *Philovenator* (Currie & Peng, 1993).

In most theropods, including the majority of paravians, the shaft of metatarsal IV is not significantly wider than those of metatarsals II and III (*e.g.*, *Deinonychus*, Ostrom, 1969; *Gallimimus*, Osmólska, Roniewicz & Barsbold, 1972; *Mononykus*, Perle et al., 1994; *Sinovenator*, Xu et al., 2002; *Tyrannosaurus*, Brochus, 2003; *Avimimus* Funston et al., 2016). *Borogovia* shares with the Late Cretaceous troodontids a notably wide metatarsal IV, which - partly owing to the relative narrowness of the shafts of metatarsals II and III - forms at least half of the mid-shaft width of the metatarsus (*e.g.*, Russell, 1969; Kurzanov & Osmólska, 1991; Currie & Peng, 1993).

The second toe of *Borogovia* is unique among non-avian theropods in showing a peculiar subset of the usual falciphoran features (Ostrom, 1969; Turner, Makovicky & Norell, 2012). Phalanx II-2 is very abbreviated, being less than half as long as phalanx II-1 (Figs. 5A–5D). As in other troodontids (*e.g.*, *Philovenator*, Xu et al., 2012; *Talos*, Zanno et al., 2011), phalanx II-2 bears a prominent ventral process (heel) extending proximally from the ventral margin of the proximal facet, which as a result is significantly larger than the distal articular surface of the same bone. Furthermore, that phalanx lacks a clear distinction between diaphysis and articular ends, a condition otherwise seen in some Late Cretaceous troodontids (*e.g.*, *Gobivenator*, Tsuihiji et al., 2014) and, in a less extreme form, in Halszkaraptorinae (*e.g.*, Cau et al., 2017). The corresponding ungual is low and robust with a flattened ventral surface bounded proximally by a low yet well-defined flexor tubercle (Figs. 5F–5H). Such morphology differs radically from the falciform and mediolaterally-compressed unguals that are widespread among paravians, including most troodontids (*e.g.*, *Deinonychus*, Ostrom et al., 1969; *Talos*, Zanno et al., 2011; *Balaur*, Brusatte et al., 2013; *Halszkaraptor*, Cau et al., 2017; *Rahonavis*, Forster et al., 2020). Although divergent from the morphology widespread among other ‘deinonychosaur-grade’ paravians, the second toe of *Borogovia* is overall similar to that of the extant bird *Casuarius* (*e.g.*, Saber & Hassanin, 2014).

*Borogovia* differs from almost all other theropods in that the phalanges of the third toe are distinctly less robust than those of the fourth toe (Osmólska, 1987). Although this feature might be a by-product of the homologous enlargement of the fourth metatarsal shared with other troodontids, the fourth toe is less robust in these taxa than in *B. gracilicrus* (*e.g.*, *Talos*, Zanno et al., 2011; *Philovenator*, Xu et al., 2012). A similar apomorphic condition is present in the parvicursorine *Mononykus* (although this taxon lacks a comparably robust metatarsal IV; see fig. 18 in Perle et al., 1994) but not in other alvarezsaurids, in which the third toe is the most robust in the foot (*e.g.*, Turner, Nesbitt & Norell, 2009).

The extensor surfaces of the phalanges in the fourth toe of *Borogovia* bear relatively shallow extensor pits with poorly defined margins (Figs. 5Q–5X). This condition is shared with the majority of coelurosaurs (*e.g.*, Ostrom, 1969; Brochu, 2003), but not with most alvarezsauroids, in which the fourth toe phalanges bear deeper extensor pits that are usually sharply bordered by the distal condyles (*e.g.*, *Monoykus*, Perle et al., 1994).

## Phylogenetic analysis

### Results of the unweighted parsimony analysis

**Figure 6:**
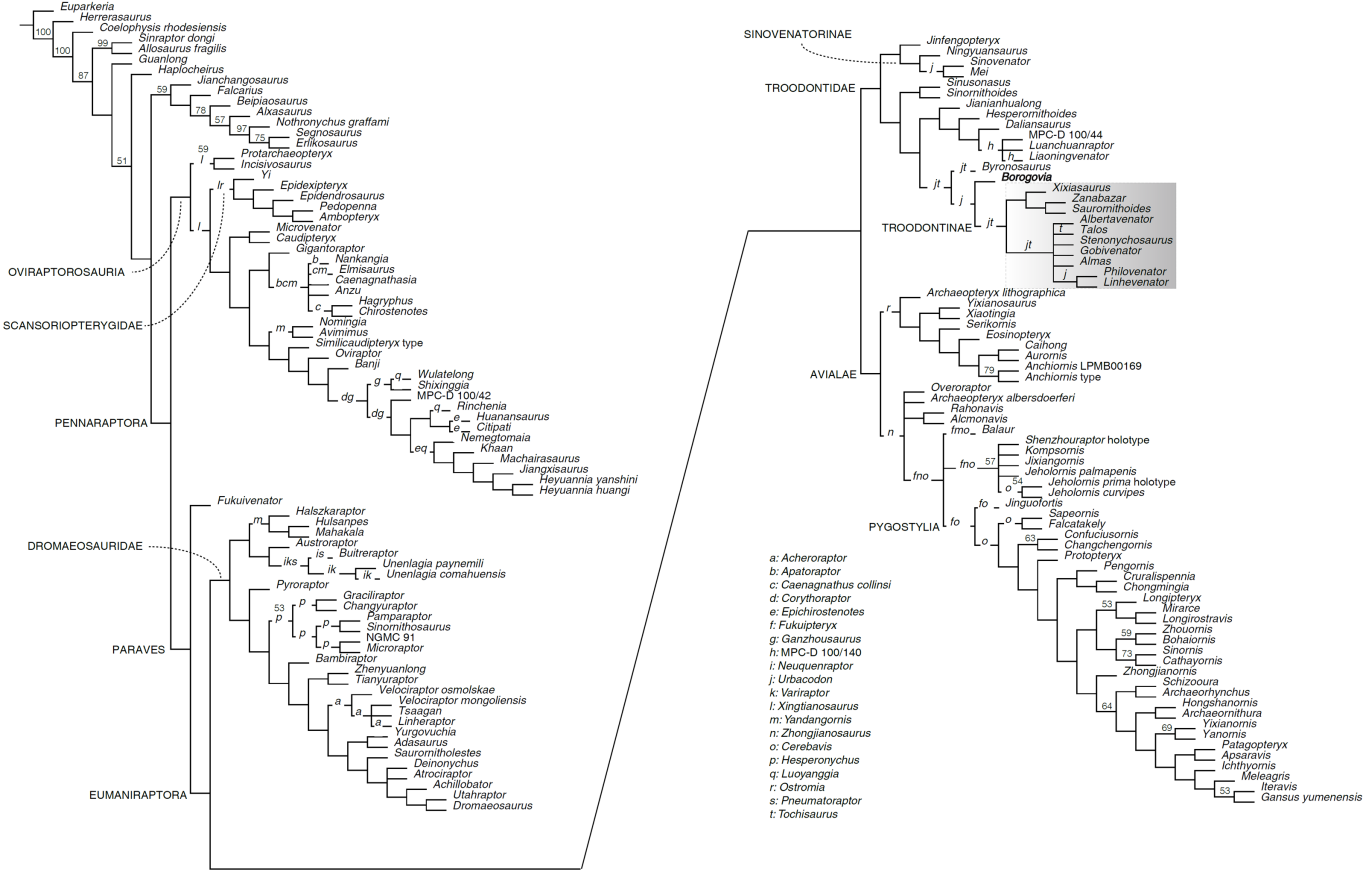
Reduced strict consensus of the shortest trees reconstructed by the unweighted phylogenetic analysis. Number at nodes indicate nodal support (jacknife values < 50, removal probability = 36). Letters at branches indicate alternative placements of the pruned OTUs relative to the reduced strict consensus tree. Grey area indicates most inclusive clade containing all alternative placements of ‘*Troodon*’ OTU resulted in preliminary analysis.

In all shortest trees found during the preliminary analysis, the very fragmentary OTU ‘*Troodon*’ was consistently reconstructed within the clade including all taxa traditionally treated as troodontids (*e.g.*, Currie, 1987; Kurzanov & Osmólska, 1991; Russell & Dong, 1993; Norell et al., 2009; Pei et al., 2021), a result that validates the use of the name Troodontidae for this branch of Paraves. In particular, our analysis supports the hypothesis that *Troodon* is a member of the most inclusive clade containing Late Cretaceous troodontids but excluding *Jinfengopteryx* and *Sinovenator*. Exclusion of the ‘*Troodon*’ OTU from the analysis does not alter significantly the tree topology, except by improving resolution among the late-diverging troodontids. The main analysis reconstructed 50,000 shortest trees of 5805 steps each (Consistency Index excluding uninformative characters = 0.2252; Retention Index = 0.5754). In both analyses, Avialae is reconstructed as the sister taxon of Troodontidae (Fig. 6). The troodontid clade shows a relatively well-resolved topology, with only a few fragmentary OTUs (*e.g.*, *Tochisaurus*, *Urbacodon*) acting as ‘wildcards’. The analysis reconstructed an early-diverging jinfengopterygine-sinovenatorine lineage, including *Jinfengopteryx* (the sole jinfengopterygine representative) and *Mei*, *Ningyuansaurus* (Ji et al., 2012) and *Sinovenator* (Sinovenatorinae). All shortest trees found in this analysis place *Borogovia* as the sister taxon to Troodontinae, which is the least inclusive group containing *Troodon* (when included), *Gobivenator*, and *Zanabazar*. Despite being relatively well-resolved, this topology is only weakly supported, which is likely due to the inclusion of very fragmentary OTUs in the analysis.

We replicated the analysis enforcing three alternative topologies reflecting the frameworks of some recent analyses of paravian relationships (Agnolín et al., 2019; Hartman et al., 2019; Pei et al., 2020). Enforcing the framework of Agnolín et al. (2019), *i.e.,* troodontids, eudromaeosaurs, microraptorines, and unenlagiines successively closer to avialans, with halszkaraptorines—not discussed in that study—set as ‘floaters’), the shortest trees reconstructed were 26 steps longer than the shortest unenforced topologies (halszkaraptorines were reconstructed as unenlagiids). Enforcing the framework of Hartman et al. (2019): *i.e.,* troodontids closer to eudromaeosaurs than the ‘Halszkaraptorinae + Unenlagiinae’ clade), the shortest trees reconstructed were 16 steps longer than the shortest unenforced topologies. Enforcing the framework of Pei et al. (2020: *i.e.,* troodontids in Deinonychosauria; *Balaur* in Velociraptorinae; *Rahonavis* in Unenlagiinae) the shortest trees reconstructed were 50 steps longer than the unenforced shortest topologies. When the latter analysis was replicated setting *Balaur* and *Rahonavis* as ‘floaters’, the shortest trees reconstructed were 12 steps longer than the unenforced shortests topologies (*Balaur* and *Rahonavis* were reconstructed as avialans).

### Results of the implied weighting parsimony analyses

The analyses produced results which variably agree with the topology reconstructed by the unweighted analysis. With *K* = 3 (most aggressive down-weighting of the homoplasious characters among the tested alternatives), the monophyly of Troodontidae as reconstructed in the unweighted analysis was not supported. In particular, the sinovenatorines (except *Ningyuansaurus*, which was placed among the basalmost oviraptorosaurs, in agreement with the interpretation of Ji et al. (2012)) were reconstructed outside Troodontidae as part of a larger clade including *Sinornithoides* and *Sinusonasus*. Additionally, the halszkaraptorines were placed as the basalmost troodontid branch, and the unenlagiines as the basalmost avialan lineage (Fig. S1 ). With *K* = 9, the large majority of the taxa reconstructed as troodontids in the unweighted analysis formed the sister group of Avialae, although *Xixiasaurus* and *Liaoningvenator* were placed closer to birds than to other troodontid-grade taxa, and *Ningyuansaurus* was placed among the basalmost oviraptorosaurus (Fig. S2 ). A similar topology, in which all troodontid-grade taxa except *Ningyuansaurus* formed a clade, was reconstructed with *K* = 15 (Fig. S3). With *K* = 27, the analysis reconstructed a monophyletic Troodontidae containing the same taxa as in the unweighted analysis (Fig. S4). A subset of the relationships reconstructed using the unweighted settings was consistently reconstructed by all iterations using Implied Weighting: the scansoriopterygids were placed in Oviraptorosauria, the microraptorines in Dromaeosauridae, *Balaur* in Avialae, and *Borogovia* among late-diverging troodontids. Several additional relationships among non-troodontid pennaraptorans supported by the unweighted analysis were also reconstructed by the analyses using Implied Weighting, with the exception of the most aggressive setting (*K* = 3): in particular, *Fukuivenator* in Paraves; anchiornithines, *Archaeopteryx*, and *Rahonavis* in Avialae; Halszkaraptorinae and Unenlagiinae in Dromaeosauridae; and *Hesperornithoides* and *Jinfengopteryx* in Troodontidae. The enigmatic *Fukuivenator* was reconstructed as a member of Paraves by the unweighted analysis, and also under all selected *K-* values except the most aggressive one. Even so, the precise placement of this taxon among Paraves needs to be treated as unsettled. The particular hypothesis that *Fukuivenator* is a very basal paravian, instead of being a dromaeosaurid, was replicated only in the Implied Weighting analysis using the least aggressive *K* value. The placements by the unweighted analysis of *Luanchuanraptor henanensis* (Lü et al., 2007) and *Ningyuansaurus wangi* (Ji et al., 2012) among troodontids were not supported by the analyses using Implied Weighting *K* values of 3, 9, and 15. The former species was reconstructed among dromaeosaurids (a placement suggested by Lü et al. (2007)), and the latter as a basal oviraptorosaur (using *K* values of 3 and 9; a placement suggested by Ji et al., 2012).

## Discussion

### Paravian tree topology and troodontid ingroup relationships

The most parsimonious topology reconstructed by our analyses places Troodontidae closer to Avialae than Dromaeosauridae, which comprises Halszkaraptorinae, Unenlagiinae, Microraptorinae, and Eudromaeosauria. The (dromaeosaurids (avialans, troodontids)) topology is supported by all iterations of the analyses using the Implied Weighting function in TNT. Although interpreted as early-diverging members of the troodontid lineage in some analyses (*e.g.*, Brusatte et al., 2014), our study supports the hypothesis that the anchiornithines are members of Avialae (see also, Agnolín & Novas, 2013; Cau et al., 2017; Pei et al., 2020). At least 19 unambiguous synapomorphies support the sister-taxon relationships between avialans and troodontids. The shortest trees in which troodontids are inferred to be closer to dromaeosaurids than birds (the ‘traditional Deinonychosauria’, with troodontids being the sister group of dromaeosaurids; Turner, Makovicky & Norell, 2012; Brusatte et al., 2014) is 12 steps longer than our ‘preferred’ topology (Pei et al., 2020, with *Balaur* and *Rahonavis* among early-diverging birds). A similar step cost is found for the ‘alternative Deinonychosauria’ scenario of Hartman et al. (2019). The positions of *Balaur* and *Rahonavis* have a significant impact on the step cost of the ‘deinonychosaurian hypothesis’ (Cau, Brougham & Naish, 2015; Forster et al., 2020): if placement of these two taxa in Deinonychosauria is enforced, the topology of Pei et al. (2020) results 50 steps longer than our ‘preferred’ scenario. Placing troodontids more rootward than dromaeosaurid-grade taxa (*e.g.*, Agnolín et al., 2019) turns out to be even less parsimonious than placing them in Deinonychosauria.

Pending a revision of the taxonomy and diversity of the Late Cretaceous troodontids from North America (see Cullen et al., 2021), our analysis supports the referral of the type material of *Troodon formosus* to the clade of paravian theropods including *Saurornithoides*, *Stenonychosaurus*, and *Zanabazar*, and traditionally named Troodontidae. Following our ‘preferred’ topology, Troodontidae includes two main subclades: a smaller one, comprising Jinfengopteryginae (represented only by *Jinfengopteryx elegans*) and Sinovenatorinae (Shen et al., 2017a), and a larger one including all Late Cretaceous taxa. It is noteworthy that a topological structure of Troodontidae very similar to that inferred in our study (*i.e.,* a basal dichotomy between the jinfengopterygine-sinovenatorine branch and the species-rich clade which includes all Late Cretaceous taxa in a relatively late-diverging position) has been recently reconstructed by Pei et al. (2021) using an independently-assembled data set. All analyses performed in the present study reconstructed *Borogovia*, despite its fragmentary nature and peculiar combination of features, as a member of the late-diverging branch including all Late Cretaceous taxa, a result which robustly validates the original interpretation of Osmólska (1987). In particular, our analyses indicate that *B. gracilicrus* is phylogenetically close to Troodontinae (*sensu* van der Reest and Currie, 2017) but might not be a member of the group.

### The taxonomic status of the Nemegt troodontids

*Borogovia gracilicrus* is known from a single specimen preserving only part of the distal portion of each hindlimb. Although fragmentary, the combination of features observable in the type of *B. gracilicrus* is clearly unique and, in particular, differentiates this taxon from all other theropods from the Upper Cretaceous of Mongolia. Osmólska (1987) discussed the possibility of synonymy among the three Nemegt troodontid taxa: *Borogovia*, the then-unnamed *Tochisaurus*, and “*Saurornithoides*” (currently *Zanabazar*) *junior* (Kurzanov & Osmólska, 1991; Norell et al., 2009). *Borogovia* lacks the markedly reduced distal end of metatarsal II which is diagnostic for *Tochisaurus* (Kurzanov & Osmólska, 1991), and is thus distinct from the latter. Although Norell et al. (2009) stated that no overlapping material between *Borogovia* and *Zanabazar* is known, the type specimens of both taxa include the distal end of the right tibia, preserved in articulation with the astragalus. It is therefore possible to make direct, though limited, comparisons in order to test the potential synonymy of *B. gracilicrus* and *Z. junior*. The astragalus of *Borogovia* differs from that of *Zanabazar* in lacking the distinct tuber and eminence present in the extensor surface in the latter taxon (Norell et al., 2009), and in the proportions and orientation of the condyles (see the ‘Differential diagnosis’). Owing to these differences, *Borogovia gracilicrus* is considered distinct from *Zanabazar junior* as well.

Additionally, based on the limited overlapping material available for *Tochisaurus* and *Zanabazar* (the proximal ends of the articulated metatarsi II to IV), Kurzanov & Osmólska (1991) distinguished *Tochisaurus* from *Zanabazar* on the basis of the more distinct anterodistal inclination of the proximal articular surface of the metatarsus (such inclination is absent in *Zanabazar*, Norell et al., 2009), and the narrower proximal end of metatarsal II relative to metatarsal IV. These two taxa are thus considered distinct as well.

All three Nemegt troodontids are reconstructed as late-diverging troodontids by our phylogenetic analyses: *Borogovia* is placed as the sister taxon of Troodontinae, *Tochisaurus* as a late-diverging troodontid of uncertain affinities (none of the alternative placements involves a direct sister taxon relationship with any of the other Nemegt troodontids), and *Zanabazar* as the sister taxon of *Saurornithoides* within Troodontinae.

### Evolution of the falciphoran condition

The falciphoran condition is characterized here as a complex of nine osteological features, outlined by Ostrom (1969) and Turner, Makovicky & Norell (2012): (1) distal articular surface of metatarsal II is markedly ginglymoid, with a distinct extensor groove (Ostrom, 1969; Norell & Makovicky, 1999); (2) distal articular surface of pedal phalanx II-1 is markedly expanded proximodorsally (Mayr et al., 2007; Turner, Makovicky & Norell, 2012); (3) pedal phalanx II-2 is no more than twice as proximodistally long as its distal trochlear eminence (Senter et al., 2004); (4) diaphysis of pedal phalanx II-2 extremely abbreviated and indistinct relative to the articular ends (Senter et al., 2004); (5) pedal phalanx II-2 bears a prominent proximoventral process (Ostrom, 1969; Makovicky, Apesteguía & Agnolín, 2005; Turner, Makovicky & Norell, 2012); (6) asymmetrical placement of the collateral grooves on pedal ungual II, with the lateral one closer to the dorsal margin than the medial one (Ostrom, 1969; Norell & Makovicky, 1999; Turner, Makovicky & Norell, 2012); (7) pedal ungual II is much larger overall than pedal unguals III and IV (Ostrom, 1969); (8) pedal ungual II is strongly curved and falciform (Ostrom, 1969); and (9) the flexor tubercle on pedal ungual II is prominent and clearly defined relative to the ventral surface (Ostrom, 1969).

Note that the falciphoran condition is the combination of several features of the whole second toe, and is not limited to the presence of a ‘sickle-shaped’ ungual alone. Although this condition is usually considered apomorphic for ‘deinonychosaur-grade’ paravians (*e.g.*, dromaeosaurids and troodontids), some of the features forming the ‘complete’ falciphoran toe are also present in other coelurosaurian groups, including in the enigmatic paravians *Balaur* and *Rahonavis* (which have been interpreted, alternatively, as avialans or dromaeosaurids, Brusatte et al., 2013; Cau, Brougham & Naish, 2015; Forster et al., 2020), in scansoriopterygids (*e.g.*, Xu et al., 2002), in *Fukuivenator* (Azuma et al., 2016), in some enantiornithines (*e.g.*, the bohaiornithids, see Wang et al., 2014) and in some extant birds (*e.g.*, *Casuarius*, Saber & Hassanin, 2014). This indicates that the falciphoran condition might be homoplastic among maniraptorans, and that at least some of the features in the second toe shared by the ‘deinonychosaur-grade’ forms could be synapomorphies of a clade more inclusive than Deinonychosauria (*i.e.,* paravian symplesiomorphies, secondarily lost among some subclades).

Unfortunately, the relationships among the pennaraptoran clades reconstructed by the alternative phylogenetic analyses published so far remain unstable and highly variable even with the inclusion of new characters and taxa (*e.g.*, Hartman et al., 2019; Cau, 2020; Agnolín et al., 2019; Pei et al., 2020; Pittman et al., 2020). The uncertainty on the relationships among the main pennaraptoran branches negatively biases any reconstruction of the sequence of evolutionary events that resulted in the origin of the falciphoran condition. Irrespective of the sequence in which the novel morphological features assembling the falciphoran condition were acquired, the phylogenetic position of *Borogovia*—consistently found to lie among the late-diverging troodontids in all analyses performed in this study—suggests that the peculiar combination of features seen in the second toe of the Nemegt taxon is a derived condition. In particular, character optimization shows that the terminal branch leading to *B. gracilicrus* is diagnosed by the loss of some of the falciphoran apomorphies shared by other troodontids. Specifically, the flattened and unrecurved shape of the second toe ungual in *B. gracilicrus* represents a specialization of this taxon and not the retention of the primitive ‘pre-paravian’ condition. Osmólska (1987) remarked that the combination of features found in the toes of *Borogovia* (in particular, the relative gracility of the third toe) is challenging to interpret from a functional perspective. As has already been shown in Dromaeosauridae (Fowler et al., 2011; Gianechini, Ercoli & Martínez, 2019), the differences in the morphology of the falciphoran toe existing among troodontids (in particular, between *Borogovia* and the other late-diverging taxa) are expected to be related to different locomotory and feeding adaptations. The phylogenetic framework proposed herein suggests that any peculiar function acquired by the foot of *Borogovia* involved the loss of previous hindlimb adaptations that are widespread among troodontids. Possibilities include loss of the predatory function of the second talon usually associated with the falciphoran condition (Ostrom, 1969; Fowler et al., 2011; Bishop, 2019), which would imply possible dietary specialization in *B. gracilicrus,* or acquisition of a peculiar locomotory adaptation (*e.g.*, an increase in cursoriality to the detriment of other functions; Holtz, 1994; Snively, Russell & Powell, 2004). Discovery of additional material of *Borogovia*, particularly craniomandibular elements, might prove helpful in reconstructing the adaptive regime that shaped its peculiar hindlimb morphology.

## Conclusions

*Borogovia gracilicrus* is a troodontid dinosaur from the Nemegt Formation of Mongolia. Although overlapping material with the other Nemegt trooodontids (*Tochisaurus nemegtensis* and *Zanabazar junior*) is limited, *Borogovia* can be differentiated from these taxa based on the unique combination of features in its tibiotarsus and metatarsus.

The phylogenetic position of *B. gracilicrus* among Maniraptora is investigated in detail for the first time. This taxon is robustly supported as a member of Troodontidae, and reconstructed among non-troodontine troodontids. These results are not biased by *a priori* assumptions on character weighting.

The foot of *Borogovia* is autapomorphic and combines troodontid synapomorphies (*e.g.*, the arctometatarsus) and the secondary loss of some elements of the falciphoran condition widespread among paravians. In particular, the morphology of the second toe of *Borogovia* might indicate an ecological niche distinct from those of other ‘deinonychosaur-grade’ paravians. Such a scenario could account for the co-occurrence of several troodontids in the paleofauna of the Nemegt Formation.

## Supplemental Information

10.7717/peerj.12640/supp-1 Supplemental Information 1 Data matrix and character list Scores of *Troodon* type OTU added separately at bottom. Character list included at bottom.

10.7717/peerj.12640/supp-2 Supplemental Information 2 Strict consensus of the shortest trees reconstructed by the weighted phylogenetic analysis (*K* = 3)

10.7717/peerj.12640/supp-3 Supplemental Information 3 Strict consensus of the shortest trees reconstructed by the weighted phylogenetic analysis (*K* = 9)

10.7717/peerj.12640/supp-4 Supplemental Information 4 Strict consensus of the shortest trees reconstructed by the weighted phylogenetic analysis (*K* = 15)

10.7717/peerj.12640/supp-5 Supplemental Information 5 Strict consensus of the shortest trees reconstructed by the weighted phylogenetic analysis (*K* = 27)

## Institutional abbreviations

MPC: Institute of Paleontology and Geology, Mongolian Academy of Sciences, Ulaanbaatar, Mongolia
ZPAL: Institute of Paleobiology, Polish Academy of Sciences, Warsaw, Poland

## Additional Information and Declarations

### Data Availability

The following information was supplied regarding data availability: The data matrix used for the phylogenetic analysis, and the character list describing the character statements scored, are available in the Supplementary File.
