# Supplementary figures and images for: The phylogenetic affinities and morphological peculiarities of the bird-like dinosaur Borogovia gracilicrus from the Upper Cretaceous of Mongolia
Source: PeerJ. 2021 Dec 6;9:e12640. doi: 10.7717/peerj.12640 (PMC8656384; doi:10.7717/peerj.12640)

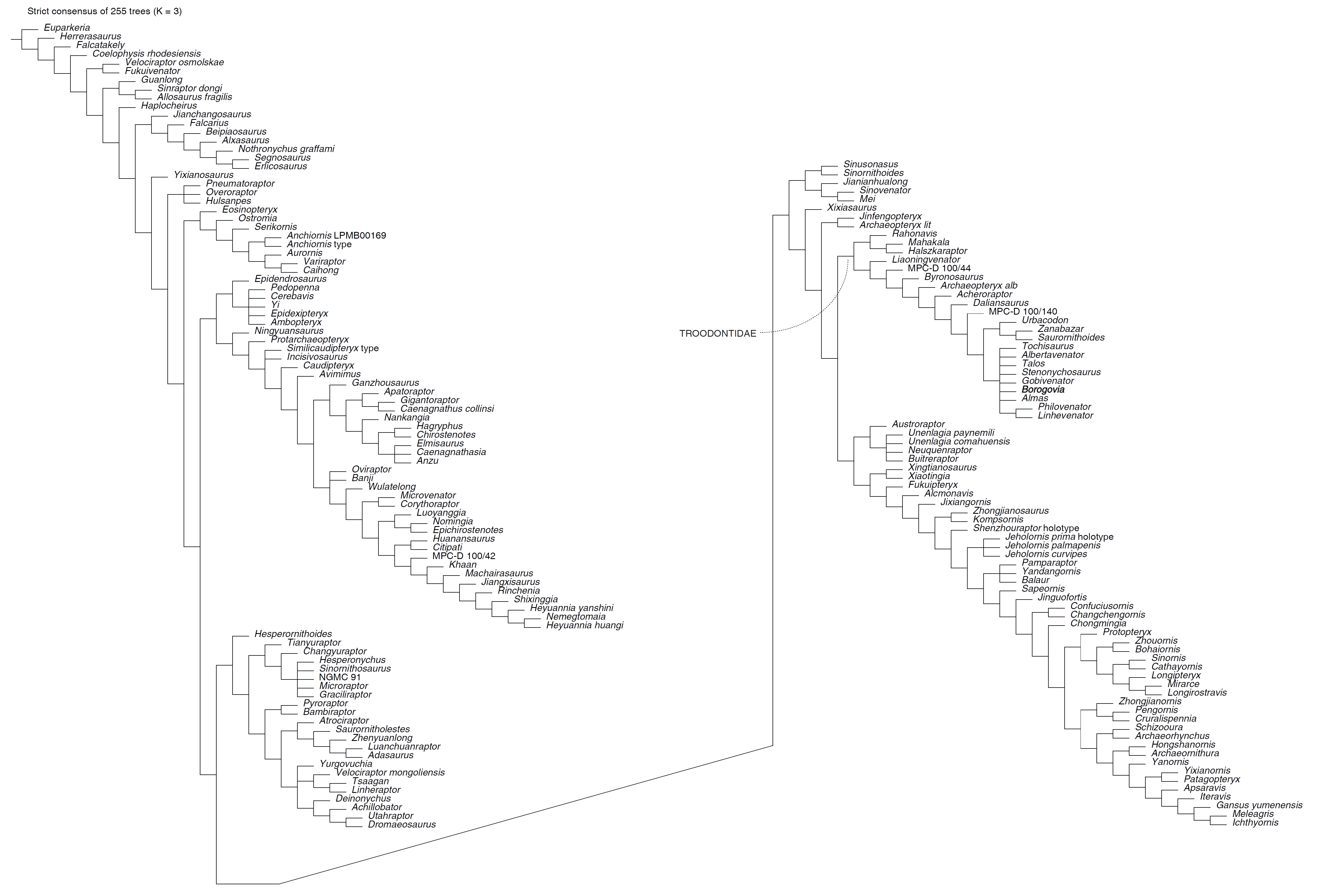

Supplement: Supplemental Information 2 [file peerj-09-12640-s002.jpg]

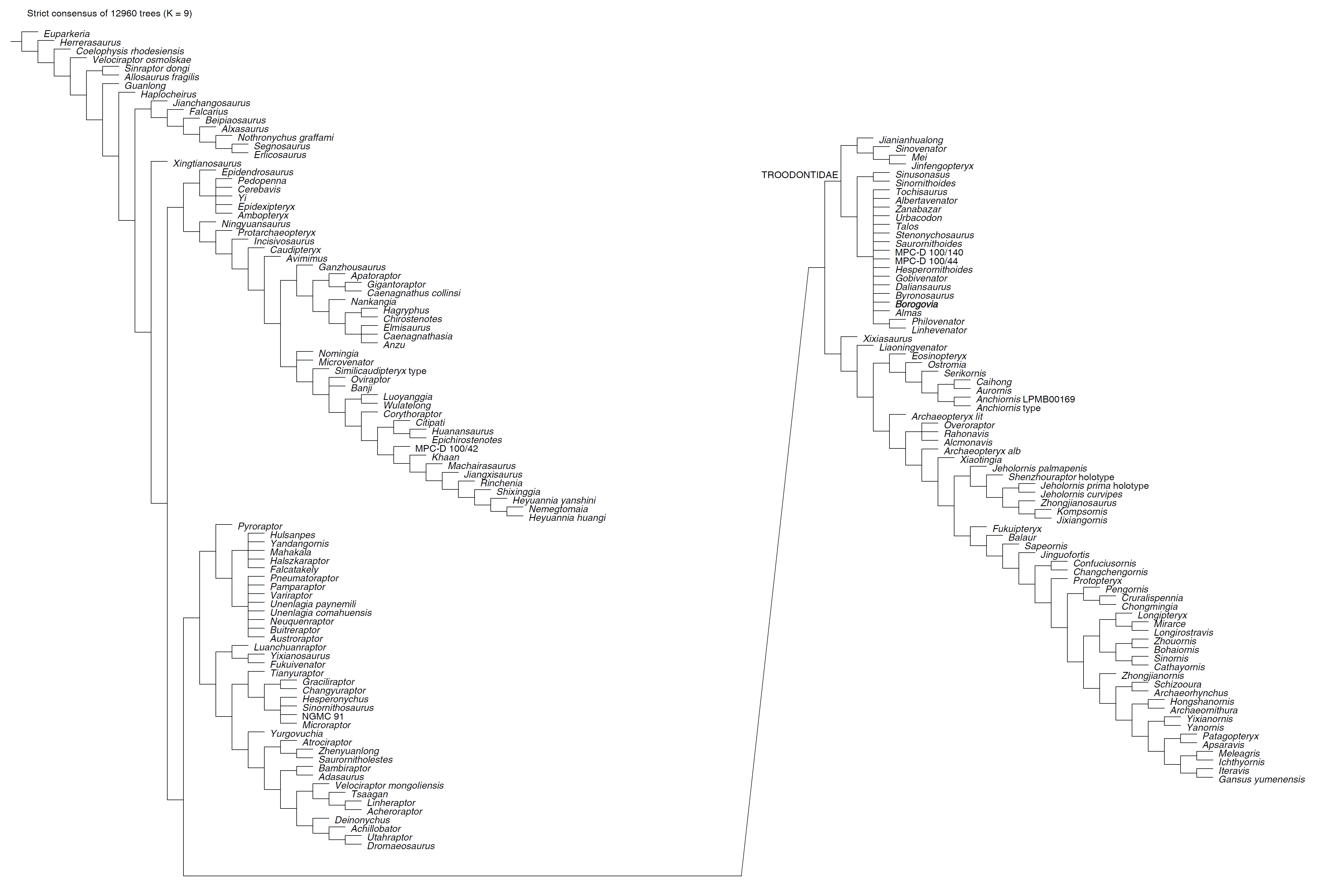

Supplement: Supplemental Information 3 [file peerj-09-12640-s003.jpg]

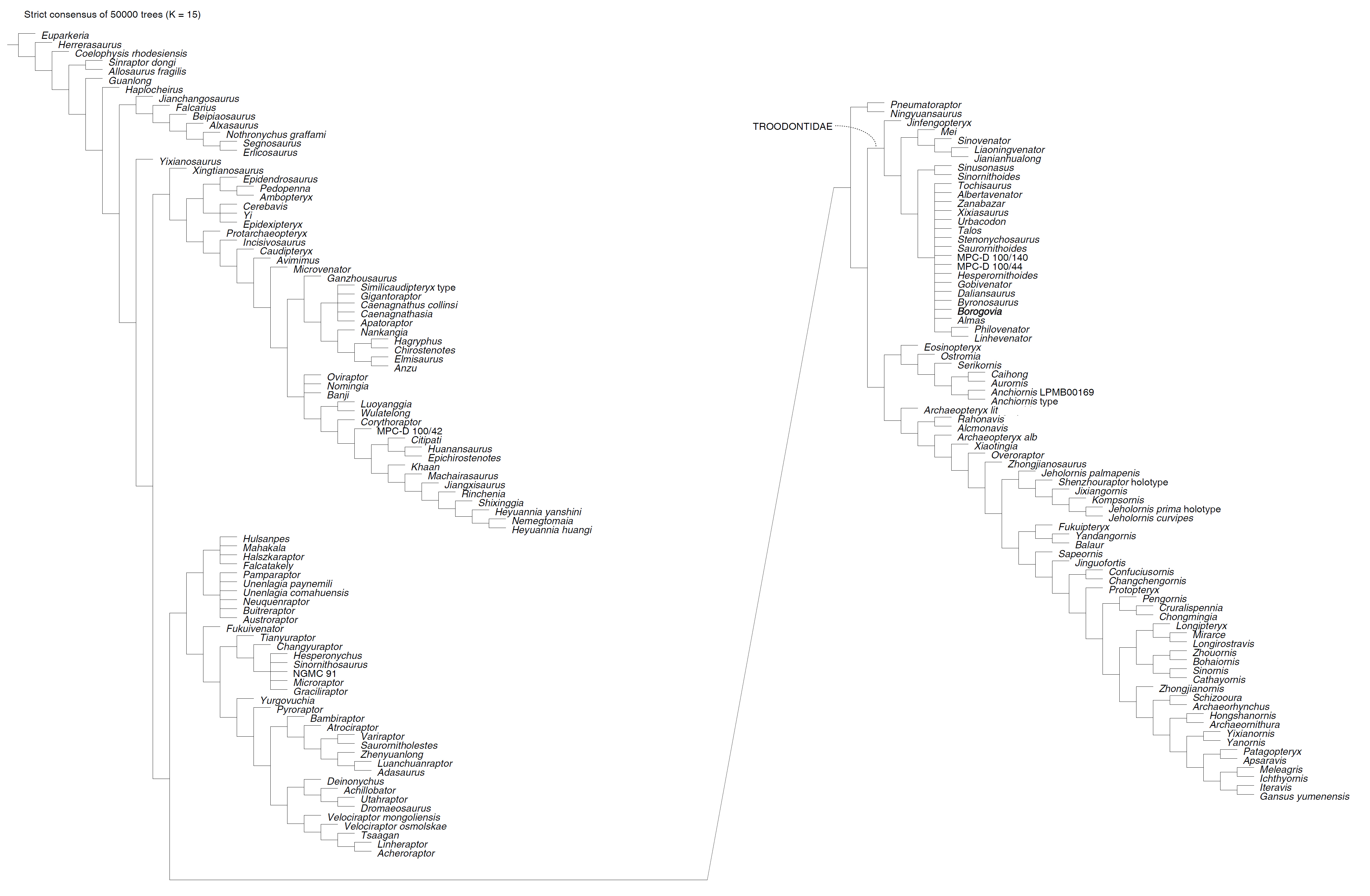

Supplement: Supplemental Information 4 [file peerj-09-12640-s004.jpg]

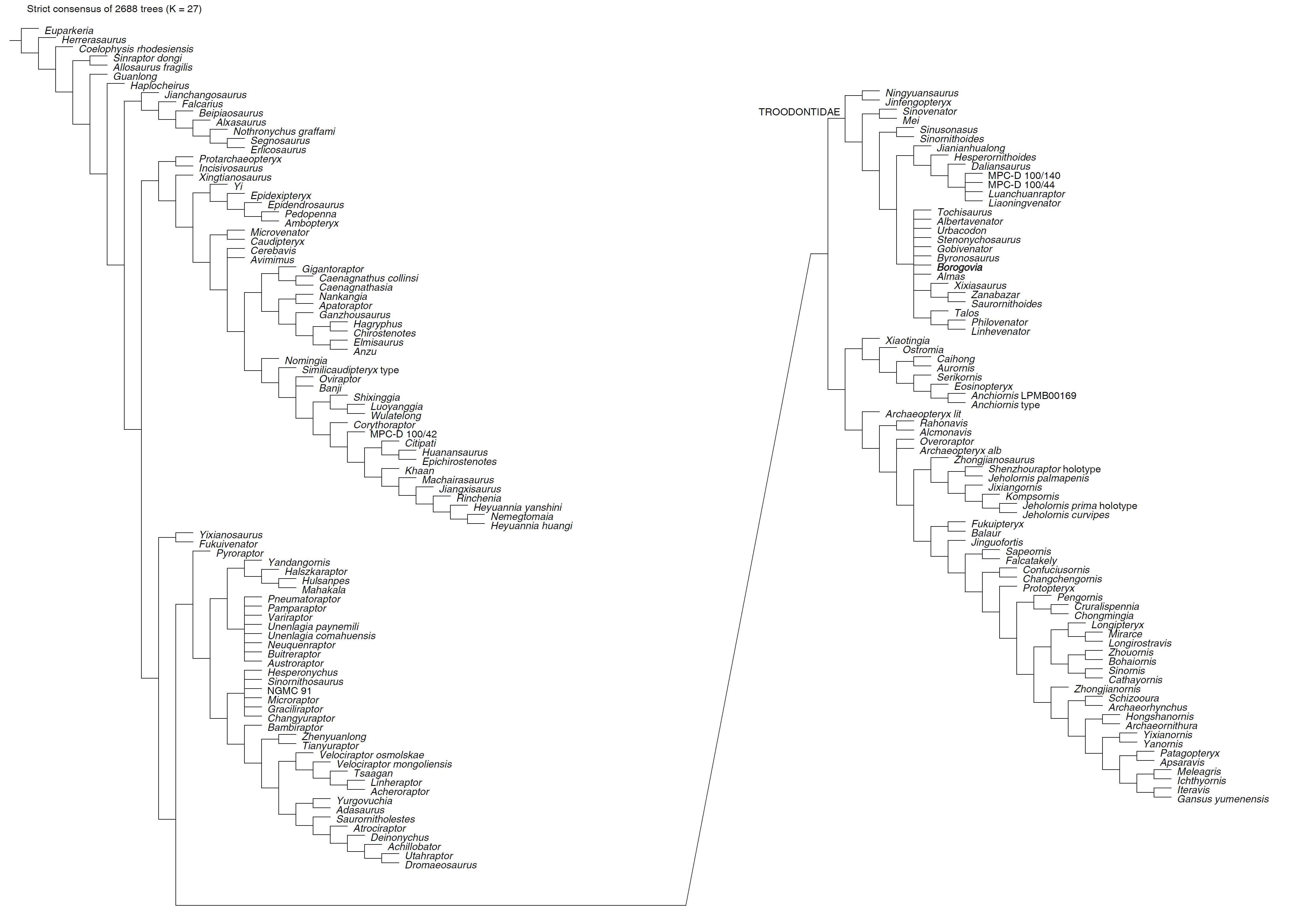

Supplement: Supplemental Information 5 [file peerj-09-12640-s005.jpg]
